# A Novel 7-Days Prolonged Dietary Deprivation Regimen Improves ALT and UA After 3–6 Months Refeeding, Indicating Therapeutic Potential

**DOI:** 10.3389/fnut.2020.00050

**Published:** 2020-05-06

**Authors:** Xiaoxue Wang, Zhihui Li, Yancong Zhao, Yaying Yu, Yanyan Xue, Chenguang Niu, Qiannan Wei, Zhijun Zhao, Shangyuan Cai, Hongxia Xu, Chenlu Zhang, Chenggang Zhang, Garrick D. Lee

**Affiliations:** ^1^The First Affiliated Hospital of Henan University, Kaifeng, China; ^2^Institute on Aging and Disease of Henan University, Kaifeng, China; ^3^Cognitive and Mental Health Research Center, Beijing Institute of Radiation Medicine, Beijing, China

**Keywords:** flexible abrosia (FA), continual dietary deprivation (CDD), alanine aminotransferase (ALT), glutamic oxalacetic transaminase (GOT), creatine kinase (CK)

## Abstract

**Objectives:** The aim of this study was to evaluate a total fasting regimen assisted by a novel prebiotic, Flexible Abrosia (FA), in more than 7 days of continual dietary deprivation (7D-CDD). Our analysis included basic physical examinations, bioelectrical impedance analysis, and clinical lab and ELISA analysis in normal volunteers.

**Methods:** Seven healthy subjects with normal body weight participated in 7D-CDD with the assistance of a specially designed probiotic. Individuals were assigned to take FA (113.4 KJ/10 g) at each mealtime to avoid possible injuries to intestinal flora and smooth the hunger sensation. During 7D-CDD, the subjects were advised to avoid any food intake, especially carbohydrates, except for drinking plentiful amounts of water. The examination samples were collected before CDD as self-control, at 7 days fasting, and after 7~14 days of refeeding. Three subjects were also tested after 6-m refeeding.

**Results:** The FA-CDD regimen significantly decreased suffering from starvation, with tolerable hunger sensations during the treatment. With the addition of daily mineral electrolytes, the subjects not only passed through the entire 7D-CDD regimen but also succeed in 12~13 days total fasting in two subjects. There was a significant reduction in blood glucose, insulin, and high-density lipoprotein levels during fasting, and the blood concentrations of uric acid (UA), alanine aminotransferase (ALT), and creatine kinase (CK) were increased. However, after more than 2 months of refeeding, the disease markers ALT, GOT, and CK either remained stable or were slightly downregulated compared to their initial D0 control level.

**Conclusion:** Our experiment has supplied the first positive evidence that, with the assistance of a daily nutritional supply of around 100 kcal total calories to their intestinal flora, human subjects were able to tolerate hunger sensations. We have found that, although 7D-CDD induced increases in UA, CK, and transferases during fasting, refeeding led the markers to become either down-regulated or unchanged compared to their initial levels. This phenomenon was further confirmed in longer-term (6 m) recovery. Our results failed to support the hypothesis that fasting induced liver damage, since ALT, GOT, and CK remained low after longer-term refeeding. Our findings indicate that the 7D-CDD regimen might be practical and that it might be valuable to design larger clinical fasting trials for improvement of health strategy-targeting in metabolic disorders.

## Background, Motivation, and Objectives

Fasting paradigms have been proved to be more effective and efficient at achieving health benefits than are other types of dietary paradigms, including dietary or calorie restriction (DR/CR) (1). Fasting in higher species has only been studied extensively as short-term or intermittent fasting schedules (IF) (2). Among various forms of IF, a recent report considered alternate day fasting (ADF), defined as strict 36-h periods without caloric intake (“fast days”) followed by 12-h intervals with *ad libitum* food consumption (“feast days”), to be one of the most extreme dietary interventions (3). This study demonstrated an improvement in markers of general health in healthy, middle-aged humans without adverse effects, even after >6 months' continuous application. Eventually, the treatment caused a 37% calorie reduction on average plus improved cardiovascular markers, reduced fat mass, improved fat-to-lean ratio, and increased beta-hydroxybutyrate, even on non-fasting days. Other applications of alternative fasting schedules have been reported that used a very low-calorie diet (200~500 kcal/day) to substitute for complete fasting for 1 to 3 weeks or a year for weight management, disease prevention, and chemotherapy facilitation (2, 4). The health improvement benefit of those paradigms was obvious. However, an individual must face months or even years of low-calorie dieting or fasting, which seemed to be less efficient and could be quite a long, uncomfortable, and inconvenient practice in human society.

It has been reported that some individuals could survive continual no-food fasting from 40 days (5, 6) or even more than a year (7, 8). Historical books from China and India also record some examples of months to years of absolutely no food status (bigu) as a way to attain longevity and spiritual distillation (9, 10). However, there was a lack of detailed records of any practical methodology and reports on measurable health-related impacts. To date, there have been three types of systematic studies related to prolonged fasting: (1) those initiated during 1950 and 1970 that created Guinness World Records of total fasting for more than a year (2, 7, 8) a series of metabolism studies led by G.F. Cahill Jr. involving prolonged starvation that led to the establishment of ketone-body metabolism during fasting (3, 11) a therapeutic fasting protocol established by O. Buchinger Jr. in 1952 that used fresh fruit or vegetable juice servings as alternative energy supplies, which led to an average total calorie intake of 200–250 kcal per day to assist an up to 21-days incomplete fasting status (4). Most other prolonged fasting studies also focused on metabolic studies and found ketone body metabolism, which eventually led to alternative fasting protocols (12, 13). There has been insufficient attempts to build up a proper, more tolerable prolonged total fasting regime as a health-improvement practice for society since the dawn of the new century due to the following concerns: (1) safety issues—whether prolonged fasting is safe for practice in human society; (2) tolerance and discomfort during the entire total-fasting procedure. In fact, during the 60~80 s of the last century, some safety concerns were reported regarding prolonged total fasting. They were usually due to extensive complete dietary deprivation. Usually, if the total fasting persisted for more than 40 days, negative consequences might start to happen (14, 15).

Based on the above historical realities, we have designed a special natural form of prebiotic intervention, Flexible Abrosia (FA, with 113.4 KJ in each 10-g pack), which consists of food-grade polysaccharose, which can be mostly absorbed by bacteria rather than the human host (16, 17). It was hypothesized that long-term DD leads gut microbiota to resort to host-secreted mucus glycoproteins as a nutrient source, which causes erosion of the colonic mucus barrier and hunger pangs (18). We have applied a daily intake of 10 g FA at three mealtimes (3 × 113.4 KJ, which is <100 kcal per day) under a fasting paradigm. The calorie supply was not targeted at maintaining basic energy supply in human; rather, it was designed to reduce the starvation of intestinal flora, which might cause injury to the human digestive system. Compared with previously reported fasting-mimicking diet (FMD, 3,000–4,600 kJ per day) facilitated-fasting paradigms (12) and Buchinger's Periodic Fasting intervention, which contained fruit juice to maintain a minimum calorie intake of 200 kcal (4), our FA-facilitated continual dietary deprivation paradigm (FA-CDD, with <100 kcal non-human-absorbable daily calorie intake) would be close to a no-calorie intake protocol. Considering the life-threatening negative consequences that usually occur after more than 40 days' complete fasting (14, 15, 19), we have suggested a practical fasting period of 1 week for the entire FA-CDD treatment. We also found that maintaining the real no food status would make the subject more efficient in overcoming the hunger sensation. Since then, the FA functional food supplement has assisted up to 1,000 volunteers to achieve the FA-CDD paradigm with tolerable sensations of hunger for a continual 7 days for the purpose of weight control or chronic disease treatment (20). Some of the volunteers voluntarily chose to extend to 14-d total fasting and experienced tolerable and favorable consequences (21).

Here, we collect seven individual subjects with typical 7D-CDD experience and focus on reporting special phenomena of and scientific evidence on the 7-D FA-CDD. Our report will supply some preliminary results of this paradigm prior to applying more sophisticated clinical trials to further evaluate the potential health improvement value of its application in human society.

## Materials and Methods

### Study Design and Participants

A complete clinical trial registration has been deposited with the Chinese clinical trial registration organization (http://www.chictr.org.cn with registration # ChiCTR-OOC-17010377). Approval of the study protocol was given by the University of Henan Human Research Protection Program under the guidance of the China Association for Ethical Studies. The protocol was also recorded with the Medical Ethics Committee of Henan Medical Association of Henan Province, and the entire clinical study was under the supervision of the ethics board of The First Affiliated Hospital of Henan University. Before initiating the program, signed informed consent was obtained before everyone participated, and history, physical, electrocardiogram, laboratory, physical, and ultrasonic exams were also performed as pre-med checks. Inclusion criteria were as follows: (i) volunteers from the hospital staff, including doctors, nurses, lab and medical technicians, etc., and their relatives, (ii) age 21–65 years, (iii) absence of any exclusionary factor among the individuals participating, such as active medical or psychiatric problems, history of heart disease and potential heart problems such as heart failure, myocardial infarction, and cardiac arrhythmia, renal dysfunction, serious blood clots, intestinal obstruction or ulcer, or type-1 diabetes patients with islet dysfunction (18).

### Procedures

Volunteers were recruited and introduced to the FA-CDD program. Before the trial started, the individuals received medical and laboratory examinations, including collections of serum, plasma, urine, and feces samples. FA-CDD involved daily oral application of a solid beverage of Flexible Abrosia (FA, Beijing Cloud Medical International Technology, Inc. China) 10 g/bag/person per treatment at three mealtimes every day on *an outpatient basis* during the fasting period. *The ingredients of FA were designed to include dietary fiber and cordyceps polysaccharide, ganoderma lucidum polysaccharide, and hericium erinaceus polysaccharide* *(**18**), which were regarded as bacteria- but not human-consumed saccharides* *(**22**). The National Food Inspection Center of China has reported the analyzed energy of 10 g FA as 113.4 KJ (27 kcal), which indicated that even if the calories from each treatment were completely absorbed by the human being, it would be* <*100 kcal daily in total, significantly less than recently reported low-calorie (500 kcal per day) intake in the treatment of cancer* *(**1**)*.

During the 7D-CDD period, individuals were advised to avoid any food intake, especially carbohydrates, and only to drink plenty of water and keep mineral electrolyte intake and vitamin supply constant. During the first 3~5 days, to overcome extreme hunger sensations and cravings for food, some individuals might consume a few pieces of fruits such as cucumber or tomato. Further, participants could also consume either 1 bag of 375 mg potassium/400 mg magnesium (DAS Gesunde Plus, Deutschland) or 1 pellet of 1.08 g (K = 10 mEq) potassium citrate extended-release tablets (Dawnrays China) every day during extreme hunger periods in the day time, which could further ameliorate fasting-induced mineral loss and reduce peristaltic pangs of the smooth muscles of the gut. According to the written informed consent forms, the individuals could quit the ongoing program at any time and at any step of the experiment without giving an explanation.

**Figure 1:**
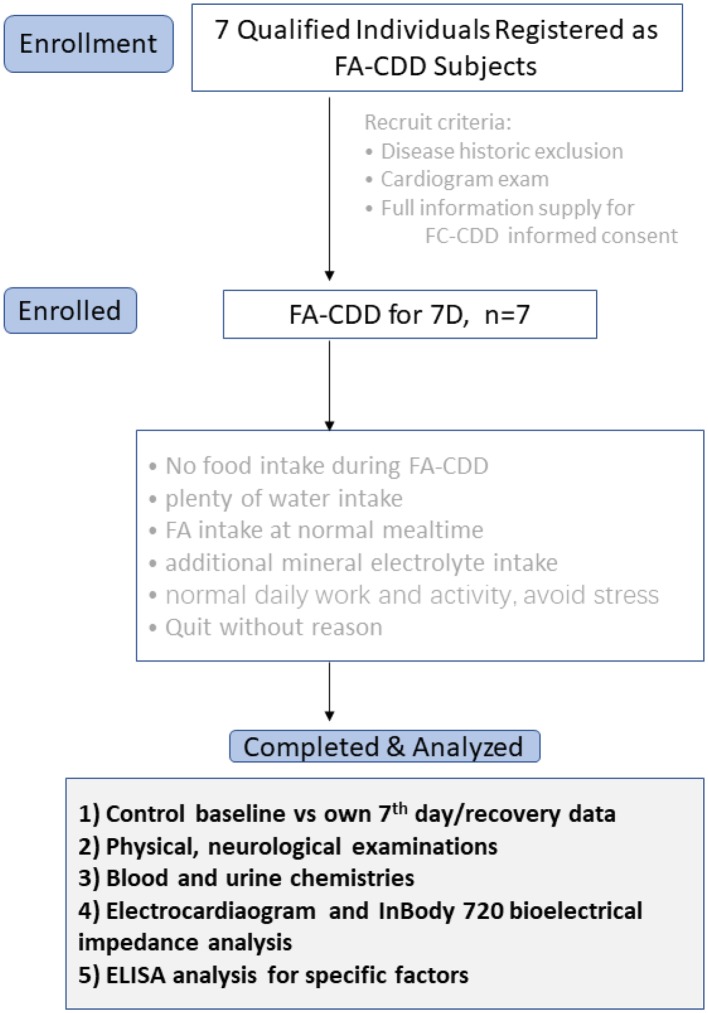
Flow chart of the clinical observation procedure.

During the entire experimental fasting period, concomitant hunger sensations and tolerance limits categorized into different levels were recorded in a questionnaire supplied by the project administration. Physical and neurological examination, weight, blood and urine chemistries, electrocardiogram, bioelectrical impedance-directed body composition analysis, and ultrasound exam were checked at three time-points by the medical authorities of the hospital: (1) before the regimen was initiated (control baseline—*ad libitum*), (2) 7th day of fasting (7D-CDD), and (3) after recovery with food intake for 7–14 days or (4) after recovery with food intake for more than 3 months to as much as 6 months (Figure 1). The medical and basic laboratory exams were performed in The First Affiliated Hospital of Henan University. The extra serum and plasma samples were collected and stored under −80°C for future molecular and mechanistic analyses. The ELISA and metagenomics tests were performed at Beijing Institute of Radiation Medicine.

Bioelectrical impedance analysis (BEIA) was performed using the InBody 720 Body Composition Analyzer at the Nutritional Department of the First Affiliated Hospital of Henan University. InBody 720 has received the approval of the FDA to analyze impedance, reactance, and resistance. The instrument expresses the relationships of water, protein, muscle, mineral, and fat content, and more, rather than just measuring Body Mass Index (BMI). The device can determine the weight of lean muscle tissue in each limb, water content, percentage body fat, mineral content, protein content, and visceral fat levels. Measurements at five time points during the CDD experiment data were supplied via a full-color print-out and were analyzed by a professional analyst at the hospital.

### Biological Sample Analysis

In addition to the routine clinical biochemical and blood/urine tests, we also performed molecular and biochemical tests on biological samples collected during the CDD experiment:

1. Plasma or serum preparations. Plasma or serum was centrifuged at 3,000 rpm and analyzed according the protocol of the hospital.
2. Serum factor measurements. TNF-α (E-EL-H1205c) and Insulin-like growth factor 1 (E-EL-H0086c) levels in serum were detected with ELISA kits (Elabscience Biotechnology Co., Ltd., Wuhan, China http://www.elabscience.cn/). The optical density was read at 450 nm using a microtiter plate reader.

### Statistical Analysis

One-way ANOVA with repeated measures was applied to data from the individuals and plotted in GraphPad Prism 8.0.1 software (GraphPad Software, Inc.). Maintaining prolonged fasting was not an easy task for all participants. To accurately reveal the effect of 7D-CDD and restrict the variation in individuals' control baselines, we used Dunnett's multiple comparisons test to evaluate the fasting and refeeding parameters by comparing them with the individual's own pre-fasting (0D-CDD) point as control. We also reported multiple comparisons between fasting and refeeding procedures. Among the seven subjects, there were two males and one female subject for whom the refeeding sample collections were achieved for more than 6 months. The individual parameters of different treatments are reported in Table 1.

**Table 1:** Physical, bioelectrical impedance (BEIA) and biochemical results under Flexible Abrosia- facilitated 7D Continual Dietary Deprivation (FA-CDD).

**Female Subjects**	**Ctrl 0D**	**Fast 7D**	**Refeed 7D**	**Ctrl 0D**	**Fast 7D**	**Refeed 7D**	**Ctrl 0D**	**Fast 7D**	**Refeed 6M**	**Control 0D**	**Fasting 7D**	**Refeed 20D**
Weight (kg)	67.20	62.10	65.50	65.10	60.10	63.40	76.40	71.40	74.10	70.20	71.40	74.10
Basal Metabolic Rate (%)	1245.62	1160.42	1251.43	1355.50	1266.93	1341.54	1351.02	1253.05	1334.68	1351.02	1253.05	1334.68
Skeletal muscle mass (kg)	21.82	19.65	21.89	25.46	23.20	24.83	24.93	22.53	24.71	24.93	22.53	24.71
Body fats (kg)	26.70	25.50	24.70	19.50	18.60	18.40	31.00	30.50	29.40	31.00	30.50	29.40
Trunk muscle mass (kg)	18.26	16.88	18.10	20.98	19.23	20.23	20.12	18.83	20.03	20.12	18.83	20.03
Body mass parameters (kg m^−2^)	26.92	24.88	26.24	26.41	24.38	25.72	29.84	27.89	28.95	29.84	27.89	28.95
**Uric Acid (μmol/L)**	**255.80**	**688.00**	**61.00**	**296.20**	**339.40**	**335.10**	**302.00**	**689.20**	**238.50**	**258.00**	**494.80**	**188.50**
**Creatine kinase U/L**	**68.00**	**121**	**92.00**	**84.00**	**96.00**	**57.00**	**87.00**	**97.00**	**96.00**	**125.00**	**192.00**	**121.00**
**Alanine aminotransferase (U/L)**	**13.30**	**17.20**	**14.00**	**70.40**	**21.80**	**45.80**	**21.70**	**143.90**	**22.10**	**25.00**	**30.50**	**18.10**
**Glutamic Oxalacetic Transaminase (U/L)**	**24.00**	**32.00**	**36.60**	**50.40**	**27.70**	**33.80**	**39.20**	**130.90**	**32.40**	**23.10**	**15.00**	**28.30**
	**3DBPS008, Male, Age 30**			**DBX009, Male, Age 45, fast 13D in total**			**3DBPS003, Male,Age 34, fast 12D in total**					
**Male Subjects**	**Ctrl 0D**	**Fast 7D**	**Refeed 14D**	**Ctrl 0D**	**Fast13D**	**Refeed 6M**	**Ctrl 0D**	**Fast 7D**	**Fast 12D**	**Refeed 16D**	**Refeed 6M**	
Weight (kg)	87.20	83.00	82.10	85.00	78.00		104.40	98.10	96.00	97.10		
Basal Metabolic Rate (%)	1690.52	1654.12	1655.27	1414.2	1351.6		1912.16	1832.55	1784.27	1870.56		
Skeletal muscle mass (kg)	35.35	34.23	34.13	36.7	34.9		41.05	39.14	37.44	39.57		
Body fats (kg)	26.10	23.60	22.60	38.3	33.8		33.00	30.40	30.50	27.60		
Trunk muscle mass (kg)	27.85	27.05	26.72	31.83	30.71		32.36	30.81	29.44	30.81		
Body mass parameters (kg m^−2^)	29.48	28.06	27.75	32.6	29.8		32.22	30.28	29.63	29.97		
**Uric Acid (μmol/L)**	**523.10**	**900.80**	**429.70**	**325.4**	**297.4**	**385.80**	**369.00**	**901.40**	**952.00**	**289.60**	**378.30**	
**Creatine kinase U/L**	**201.00**	**196**	**157**	**145**	**165**	**120**	**259.00**	**297**	**267**	**191**	**203.00**	
**Alanine aminotransferase (U/L)**	**145.40**	**176.90**	**85.60**	**19.00**	**28.50**	**18.40**	**57.40**	**48.90**	**43.60**	**65.10**	**52.70**	
**Glutamic Oxalacetic Transaminase (U/L)**	**74.00**	**97.00**	**34.70**	**36.10**	**32.10**	**28.90**	**33.50**	**32.30**	**33.30**	**35.60**	**25.10**	

*Blood sample collection and BEIA tests were performed under different timing schedules for subjects DBB1020 and 3DBPS003*.

*Therefore, the timing points of biochemical examination (in bold font) and BEIA were not the same*.

*Subjects 3DBPS003 and DBX009 automatically experienced 12D and 13D total fasting and accepted biochemical examination after 54 d and 6 m refeeding recovery, respectively*.

## Results

### Physiological and Bioelectrical Impedance Analysis

Seven subjects successfully accomplished a 7D-CDD trial. Among them, both DBX009 and 3DBPS003 voluntarily experienced 13D and 12D total fasting under the assistance of FA and strict medical monitoring. During the experiment, the subject's physical experience was recorded in a diary table supplied by the project organizers, and their medical conditions were strictly monitored by hospital medical experts. Bioelectrical impedance analysis (BEIA) indicated that the body weight (BW) and body mass parameters reduced moderately during 7D-CDD (1~2 lbs. per day) and that the refeeding recovered these parameters at a reasonable rate (Table 1). Meanwhile, the basal metabolic rate (BMR) had reduced to a lower speed at 7D fasting and recovered after refeeding (Table 1). However, it seems that muscle-related parameters (skeletal muscle mass and trunk muscle mass) recovered faster than fat-related parameters (Body fats and Visceral fat area) during refeeding (Table 1). These results might imply that the human under fasting may utilize more fat than muscle after longer-term fasting, which might explain the slow drop in BW during total fasting (Table 1).

### The Different Metabolic Patterns of Lipid and Protein During and After Fasting

**Figure 2:**
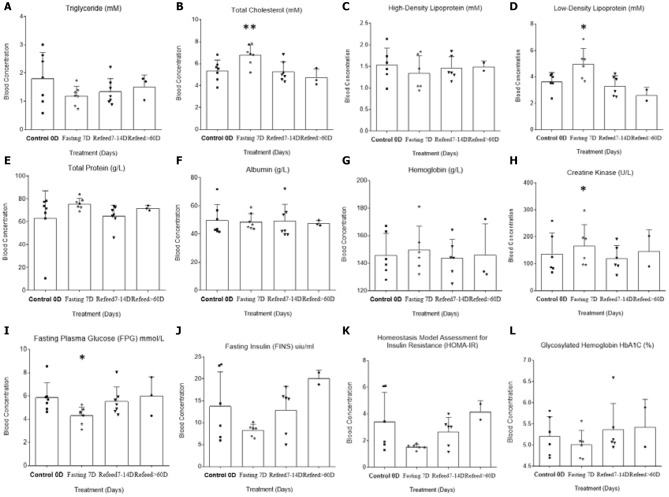
Differential analysis among 0D, 7D fasting, and refeeding results from clinical laboratory tests. All differences in 0D vs. 7D fasting and either 15D or more than 2~6 months' refeeding (three subjects donated biological samples over a longer recovery period) were analyzed. X-axes refer to the control 0D, fasting 7D, and refeeding 7–14D and >60D groups, respectively. Graph were created with GraphPad Prism 8.0.1 software using one-way ANOVA with repeated measures, and the dots around each column represent the actual values of group subjects on the specific treatments. The larger ^*^ symbol above the Fasting 7thD column indicates statistical significance (^*^p < 0.05, ^**^p < 0.01; the actual values are represented in the manuscript).

Based on the different patterns of fat and protein indicated by BEIA, we further tested and analyzed blood biochemical lab results. At 7D fasting, there were increases in both total cholesterol and low-density lipoprotein (LDL-“bad”) levels and decreases in triglyceride and high-density lipoprotein (HDL-“good”), which confirmed previous reports in cleansing cholesterol during fasting (Figures 2A–D, Total Cholesterol *F* = 10.67, *p* = 0.0054; LDL *F* = 9.621) (23). These results indicated the possibility of increased consumption of lipid-related energy supply (triglyceride and HDL) to support ketone bodies while enhancing side product clearance during autophagy of unhealthy tissue during starvation.

Meanwhile, although the protein metabolism levels tested showed a relative increase at 7D fasting, they were either slightly decreased or stabilized after refeeding (Figures 2E–H, Creatine Kinase *F* = 6.27, *p* = 0.021). The pattern of action in albumin (Figure 2F, *p* > 0.05), which free fatty acids attach and transport throughout the body for the alternative energy supply, might indicate activation of alternative energy supply from ketone metabolism. Accordingly, blood triglyceride levels, which involve fatty acid delivery via albumin, were decreased during fasting and quickly returned to control (Figure 2A, *P* < 0.5, limited sample size). Regarding carbohydrate metabolism, both glucose and insulin levels were down-regulated during 7D fasting as expected (Figures 2I–L). Prolonged fasting decreased insulin resistance (HOMA-IR), which was related to an increase in insulin sensitivity (Figures 2I–K, Glucose *F* = 8.015, *p* = 0.0243). Glycated hemoglobin (HbA1C), a form of hemoglobin used in clinic to identify the 3-month average plasma glucose concentration, remained unaffected during fasting and even after more than 2 months of recovery (Figure 2L, *p* > 0.5).

### Tissues With Unhealthy Status Seem to Be Preferentially Eliminated During Prolonged Fasting

**Figure 3:**
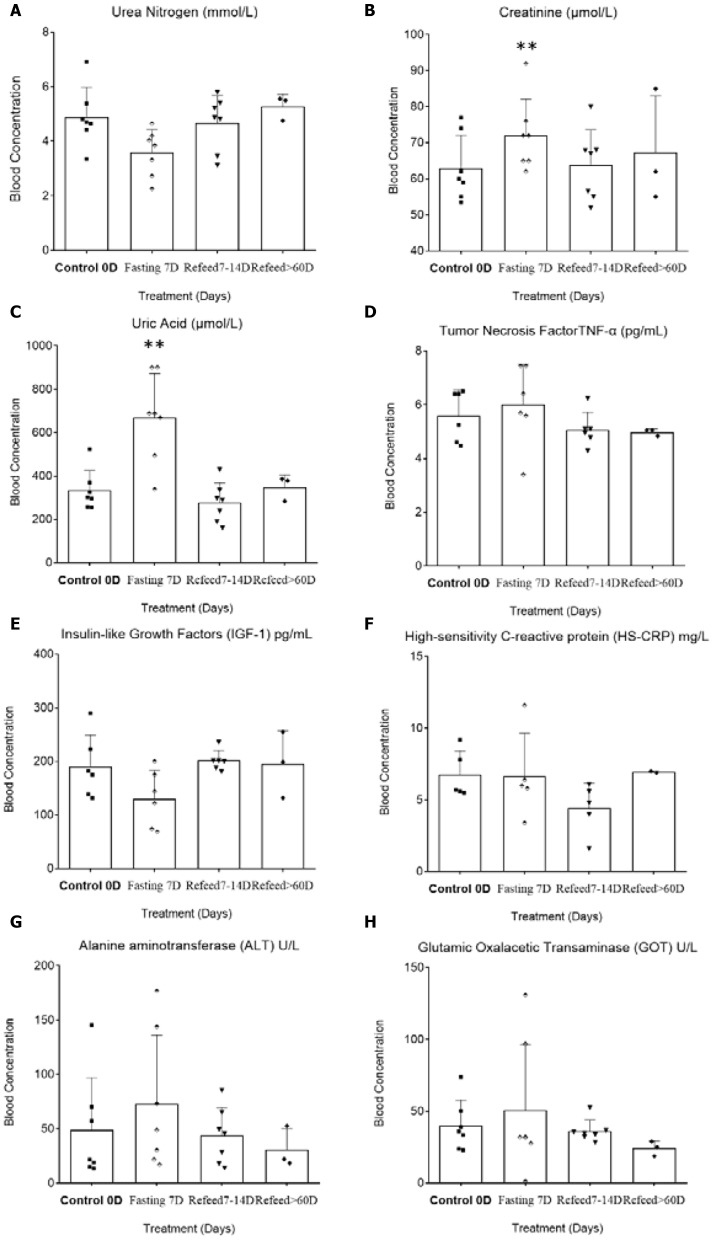
Differential analysis among 0D, 7D fasting, and refeeding results from either clinical laboratory tests or ELISA detection. All differences in 0D vs. 7D fasting and either 15D or more than 2~6-months refeeding (three subjects donated biological samples after 6 months) were analyzed. For the specific ELISA analysis, there was a lack of results from further refeeding experience. X-axes refer to control 0D, fasting 7D, and refeeding 7-14D and >60D groups, respectively. The graph was created with GraphPad Prism 8.0.1 software using one-way ANOVA with repeated measures, and the dots around each column represent the actual values of group subjects on the specific treatments. The larger ^*^ symbol above the Fasting 7thD column of **(B)** and **(C)** indicates statistical significance (^*^p < 0.05, ^**^p < 0.01; and the actual values are represented in the manuscript).

While 7D-CDD induced a reduction in blood urea nitrogen (BUN), this prolonged fasting effectively increased both creatinine (Cr) and uric acid (UA) at 7D CDD (Figures 3A–C). Except for the markers of kidney function indicators, increase in daily Cr excretion, a breakdown byproduct in muscle metabolism, is usually related to high-protein diets, and a decrease in BUN may indicate the operation of protein recycling procedures (24). Besides, long-term fasting efficiently enhanced both UA and Cr blood levels, even under limited sample size, and these returned to normal levels, as reported previously (1) (Figures 3A–C, BUN *p* = 0.085; Creatinine *F* = 7.14, *p* = 0.0093; UA *F* = 28.62, *p* = 0.0016). In addition, both Alanine Aminotransferase (ALT) and Glutamic Oxalacetic Transaminase (GOT) showed a tendency to increase during fasting, as previously reported, but we found that the recovery from prolonged fasting led to a decrease in the tendency, though without statistical significance due to the limited sample size (Figures 3G,H). Correspondently, the results from creatine kinase (CK) also showed an increase during 7D-CDD (Figure 2H). CK is an enzyme found in the heart, liver, brain, and skeletal muscle. A higher level of CK usually indicates tissue damage, which releases it into circulation, and is regarded as a biomarker of heart failure, myocardial injury, or liver damage in clinic. In the unhealthy subjects whose markers showed abnormal liver or heart function, 7D prolonged fasting caused a higher increase in concentration. Longer-term recovery levels of ALT, GOT and CK from 7D-CDD have shown lower than control levels in most of the subjects. The results indicated that, after the subject recovered from 7D fasting, the values of each unhealthy subject tended to return at a level that was lower than prefasting control (Table 1).

Additionally, results from Insulin-like growth factor type I (IGF-I), tumor necrosis factor (TNF-α), and C-reactive protein (HS-CRP) showed significant changes during 7D fasting but returned to normal after refeeding. However, there was a lack of significance due to the limited sample size (Figures 3D–F, *p* > 0.5).

## Discussion

Our 7D FA-CDD paradigm has been demonstrated to be a more tolerable and efficient regimen and is the most practical for long-term total fasting practice. The subjects' personal experience records indicated that, under the assistance of FA with proper mineral supply at every mealtime, subjects were more able to tolerate hunger sensations, with fewer pangs. Under the recommendation of drinking plenty of water to speed up the cleansing of metabolic wastes and sufficient mineral supply such as potassium and magnesium to release spasms of the smooth muscle of the digestive system, subjects experienced reasonable body weight decrease (about 1~2 lb per day) plus moderate hunger sensations. In addition, the speed of body-weight reduction was moderate, which might be safely buffered and protected without showing dramatic dehydration, as in some current commercial body-weight reduction programs.

Of the factors tested, we found that triglyceride, HDL, glucose, insulin, BUN, and IGF-1 showed tendencies to decrease. On the other hand, cholesterol, LDL, total protein, hemoglobin, lactate dehydrogenase, creatinine, uric acid, TNF-α, Cr, CK, ALT, and GOT all showed tendencies to increase (Figures 2, 3). The factors that were up-regulated during the long-term fasting seemed to be related to life-critical and beneficial survival nutrition, which the system would conserve sparingly. Those factors that were down-regulated during fasting were usually related with either alternative energy supply (such as total protein and hemoglobin), reserved system consumption (such as BUN, triglyceride, and IGF-1), or negative factors such as cholesterol, LDL, UA, TNF-α, ALT, GOT, and CK. It seems that the system automatically chose the beneficial and critical factors to reserve and selected the harmful elements to eliminate during total fasting, which might indicate that prolonged fasting-related autophagy is preferentially targeted to damaged or unhealthy tissue.

Fatty acid oxidation disorders (FAODs) has been reported to lead to deficient energy production and intermittent symptoms through increased β-oxidation, which may occur after 48 h of fasting in adults (25). Therefore, previous evidence assumed that 48-h long-term fasting may cause injury to liver function. Choline-deficient diets were reported to cause hepatic dysfunction and steatosis. However, most of the previous so-called prolonged fasting studies were related only with short-term animal models (either alternate day fasting or a shorter fasting period of <3 days). There was a lack of evidence for liver injury being induced by real prolonged fasting for more than 5 days in human. In fact, other clinical studies indicated that prolonged fasting only modestly diminished plasma choline but was not associated with signs of choline deficiency, such as perturbed lipoprotein secretion and liver damage (26). Our results indicated that the changes in ALT, GOT, and CK after 7D or longer CDD might be quite beneficial, especially in our three subjects providing 6-months refeeding recovery data (Table 1). It seems that, along with the fully metabolized fat tissues to ketone body metabolism, it might start to clean up any potential harmful metabolize, which created a healthier environment to the liver or myocardial function (unpublished results).

## Conclusion

Ketogenic or very-low-carbohydrate diets favor mitochondrial respiration for energy metabolism by imitating the fasting process (27). Wei tested the effects of a fasting-mimicking diet (FMD) associated with aging and age-related diseases and demonstrated that three FMD cycles could reduce BW and trunk and total body fat (12). If we could apply some specific period of ketone-generating food such as a low-carbon diet in the refeeding session after our long-term CDD regimen, we could expect a significant reduction in gain of body weight in fat and resolve the critical concern of such a fasting regimen in longer-term practice. In that way, we would be able to apply such a paradigm for treating metabolic syndrome. Either the prolonged fasting could be more efficient in treating the chronic disease, or metabolic syndrome needs to be confirmed in more detail by larger, more strict clinical trials with longer-term analysis.

## Data Availability Statement

All datasets for this study are included in the article, and further inquiries can be directed to the corresponding author Garrick D. Lee at Garricklee@biomed-sci.ac.cn.

## Ethics Statement

The studies involving human participants were reviewed and approved by University of Henan Human Research Protection Program was under the guidance of China Association for Ethical Studies. The patients/participants provided their written informed consent to participate in this study.

## Author Contributions

GL and CgZ have designed the study and the trial. GL, XW, HX, ZZ, SC, and CN fully managed and performed the trials. YZ, ZL, YX, and QW managed clinical laboratory analysis. YY and ClZ performed InBody 720 body composition analysis. ZL, QW, and YX performed ELISA and other blood tests. GL wrote and revised the paper.

## Conflict of Interest

The authors declare that the research was conducted in the absence of any commercial or financial relationships that could be construed as a potential conflict of interest.
